# Linalool prevents oxidative stress activated protein kinases in single UVB-exposed human skin cells

**DOI:** 10.1371/journal.pone.0176699

**Published:** 2017-05-03

**Authors:** Srithar Gunaseelan, Agilan Balupillai, Kanimozhi Govindasamy, Karthikeyan Ramasamy, Ganesan Muthusamy, Mohana Shanmugam, Radhiga Thangaiyan, Beaulah Mary Robert, Rajendra Prasad Nagarajan, Veeramani kandan Ponniresan, Pierson Rathinaraj

**Affiliations:** 1 Department of Biochemistry and Biotechnology, Annamalai University, Annamalainagar, Tamilnadu, India; 2 Department of Biochemistry, Dharumapuram Gnanambigai Government Arts College for Women, Mailaduthurai, Tamilnadu, India; 3 Centre for Transdisciplinary Research and Innovation- Wintec Private Bag, Waikato Mail Centre, Hamilton, New Zealand; University of Alabama at Birmingham, UNITED STATES

## Abstract

Ultraviolet-B radiation (285–320 nm) elicits a number of cellular signaling elements. We investigated the preventive effect of linalool, a natural monoterpene, against UVB-induced oxidative imbalance, activation of mitogen-activated protein kinase (MAPK) and nuclear factor kappa-B (NF-κB) signaling in HDFa cells. We observed that linalool treatment (30 μM) prevented acute UVB-irradiation (20 mJ/cm^2^) mediated loss of activities of antioxidant enzymes in HDFa cells. The comet assay results illustrate that linalool significantly prevents UVB-mediated 8-deoxy guanosine formation (oxidative DNA damage) rather than UVB-induced cyclobutane pyrimidine (CPD) formation. This might be due to its ability to prevent UVB-induced ROS formation and to restore the oxidative imbalance of cells. This has been reflected in UVB-induced overexpression of MAPK and NF-κB signaling. We observed that linalool inhibited UVB-induced phosphorylation of ERK1, JNK and p38 proteins of MAPK family. Linalool inhibited UVB-induced activation of NF-κB/p65 by activating IκBa. We further observed that UVB-induced expression of TNF-α, IL6, IL-10, MMP-2 and MMP-9 was modulated by linalool treatment in HDFa cells. Thus, linalool protects the human skin cells from the oxidative damages of UVB radiation and modulates MAPK and NF-κB signaling in HDFa cells. The present findings substantiate that linalool may act as a photoprotective agent against UVB-induced skin damages.

## Introduction

The skin is the largest organ of the body and serves as the barrier between the environment and internal cellular milieu which determines its critical function in the preservation of body homeostasis, and eventually organism survival [1]. As skin is continuously exposed to numerous biotic and abiotic factors, it has been evolved with protective mechanisms in order to cope up local and global aggressive environment [2]. For example, skin possesses strong antioxidant systems which maintain redox homeostasis against oxidative threat in the cellular milieu [3]. Further, recently Solmonski described the role of neuroendocrine systems such as melatonin/serotonin in the maintenance of cellular homeostasis in the skin against various environmental stresses [4]. Ultraviolet radiation (UVR) is the prominent environmental agent which continually affects cellular homeostasis in the human skin [5]. Solmonski et al. (2014) reported significant alterations in the neuroendocrine system after UVB exposure correlated with carcinogeneic events in the skin cells [6]. In addition, UVB stimulates cortisol production in the human skin keratinocytes and melanocytes which are predictable implications in local cancerogenesis [7]. It has also been well reported that ultraviolet -B (UVB; 285–320 nm) alters skin homeostasis through oxidative imbalance and induces several adverse effects such as erythema, edema, inflammation, photoaging and skin cancer [8].

UVB exposure induces reactive oxygen species (ROS) which activates several cellular signaling events [9]. Mitogen-activated protein kinases (MAPKs), a group of serine/threonine protein kinases, are reported to be activated by UVB-mediated ROS generation. Several important MAPKs involved in cellular signaling which includes extracellular signal-regulated kinases (ERK1/2), c-Jun N-terminal kinase (JNK) and p38 kinases [10]. Activation and subsequent phosphorylation of protein kinases are important in the regulation of cellular functions, such as differentiation, proliferation, apoptosis, inflammation and activation of activator protein-1 (AP-1) and nuclear factor-kappa B (NF-κB) [11].

Inflammatory responses after UVB-induced DNA damage was a common phenomenon in UVB-irradiated skin. UVB-induced DNA damage triggers NF-κB, a redox sensitive transcriptional factor, dependent secretion of inflammatory cytokines in dermal cells thus leading to apoptosis [12]. NF-κB upon activation by UVB radiation translocated to the nucleus where it mediates transcriptional activation of TNF-α, IL-6, IL-10 and COX-2 expression [13]. Moreover, NF-κB regulates matrix metalloproteinases (MMPs) which are involved in the deprivation of extracellular matrix and collagen remodeling [14]. Further, UVB-mediated NF-κB specifically binds at p53 promoter region and induces apoptosis [15]. Thus, inhibition of UVB-mediated oxidative DNA damage and subsequent NF-κB activation might prevent inflammation, photoaging and apoptosis in human skin cells.

Skin cells exhibit effective safeguarding network by inducing antioxidants activity. The ROS generated by UVB radiation can overcome cellular antioxidants network and mediate significant damages to cutaneous cells [16]. UVB-mediated ROS generation depletes enzymatic and nonenzymatic antioxidants of the skin and modifies DNA, lipids and proteins leading to skin cell apoptosis [17]. In order to reduce the risk of adverse effects from UV irradiation, considerable attention has been paid through implementation of novel prevention approaches. It is proposed that the use of antioxidant derivatives may prevent premature skin ageing and other skin adverse effects [17]. Several natural antioxidants exhibit beneficial effects against UVB radiation induced cellular and molecular changes [12,18]. Melatonin, a natural defense system, present in the skin possesses anti-apoptotic and anti-carcinogenic effects by protecting nuclear and/or mitochondrial DNA from UVB-induced oxidative damage or acting as electrons donor(s) to selected proteins [19–20]. Hence, it is hypothesized that natural antioxidants could scavenge UVB-induced ROS and subsequent cellular signaling in the exposed skin cells.

Linalool is a monoterpene antioxidant, volatile oils of various aromatic species, used in Indian medicine systems. Linalool possesses strong antioxidative property and scavenges ROS generated by various toxicants [21]. Furthermore, linalool shows anticancer property in tumor cells [22], and it induces apoptosis in human leukemia cells [23]. However, its effect against UVB-radiation mediated oxidative DNA damage and its subsequent molecular events have not been adequately investigated. The present study investigates the protective effect of linalool against UVB-induced DNA damage, inflammatory signaling, photoaging and apoptotic events in HDFa cells.

## Results

### Linalool prevents UVB-induced cytotoxicity and ROS generation in HDFa

**Fig 1:**
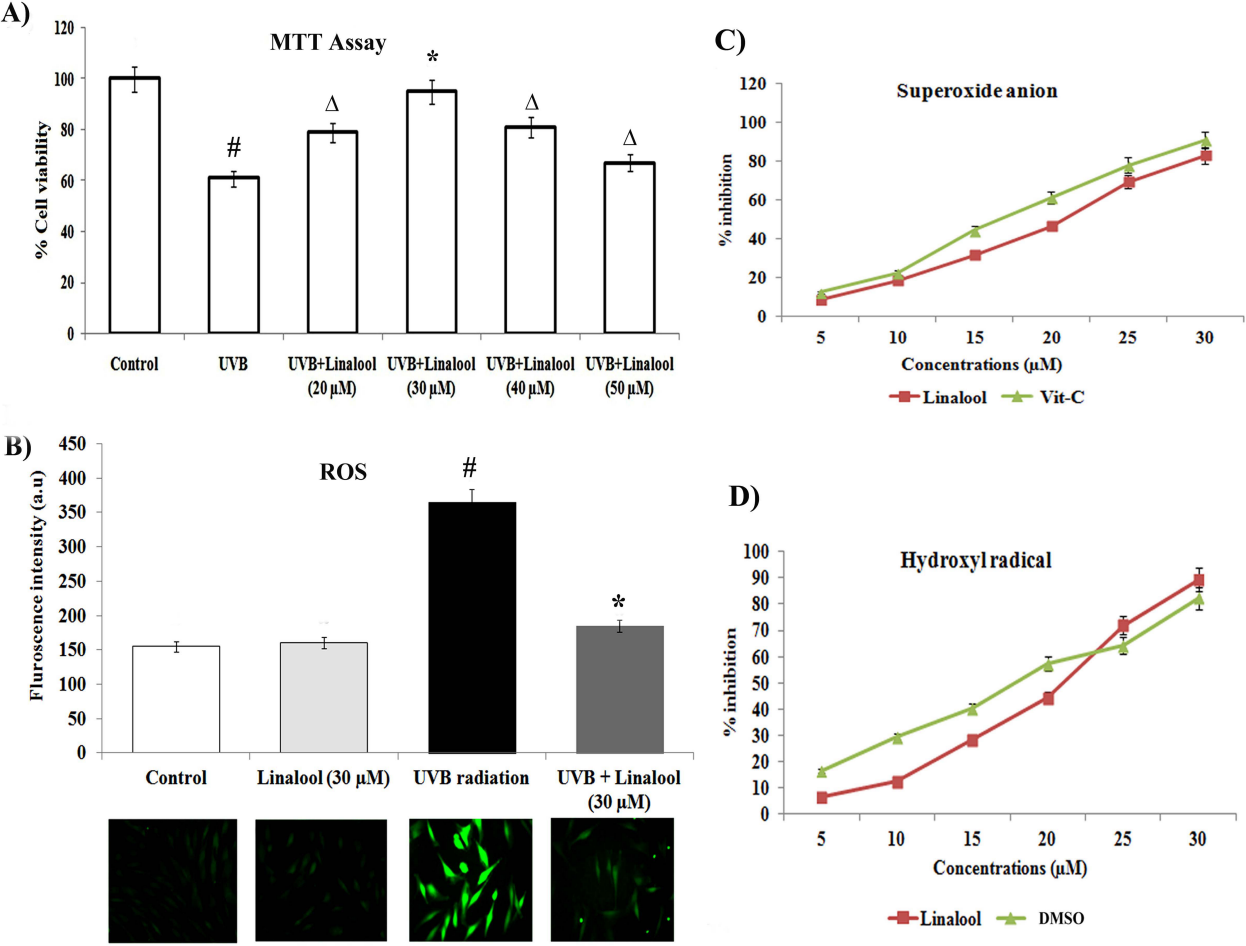
A. Cytoprotective effect of linalool on UVB-irradiated HDFa cells. The cells were treated with different concentrations of linalool (20–50 μM) and UVB-irradiated then cell viability was determined by MTT assay after 24 h incubation. Data expressed as means ± SD of six experiments in each group. Values not sharing a common marking (#,* and Δ) differ significantly at P < 0.05 (Duncan’s multiple-range test). B. The effect of linalool on UVB-induced ROS generation (DCFH-DA staining) in HDFa cells. ROS generation was examined with a fluorescence microscope (Cell Imaging Station, Life Technologies) under a green lamp (original magnification, 20X). Bar diagram represents fluorescence intensity made with excitation and emission at 485 ± 10 and 530 ± 12.5 nm respectively, using a multimode reader (Teccan, Austria). Data expressed as means ± SD of six experiments in each group. Values not sharing a common marking (# and *) differ significantly at P < 0.05 (Duncan’s multiple-range test). C&D. Free radical scavenging activity of linalool against superoxide radical and hydroxyl radical scavenging activity. Values are given as means ± SD of six experiments in each group.

To assess the cytoprotective effect of linalool against UVB toxicity, we carried out MTT based cytotoxicity assay in HDFa cells. In this study, single low dose UVB-exposure (20 mJ/cm^2^) induced significant cytotoxicity. Conversely, linalool (30 μM) pretreatment significantly prevented UVB-induced cytotoxicity in HDFa cells (Fig 1A).

Free radical scavenging capacity of compounds is related to its antioxidant activity. In this study, we tested the free radical scavenging activity and antioxidant property of linalool using in vitro free radical scavenging assays. Fig 1C and 1D shows the superoxide anion and hydroxyl radical scavenging ability of linalool. Linalool inhibits hydroxyl radicals and superoxide anion formation in a dose dependent manner. The IC_50_ value of linalool on superoxide anion scavenging activity was 21.2 ± 0.04 μM, and hydroxyl radical scavenging ability was 27.5 ± 0.02 μM, respectively.

UVB radiation generates intracellular ROS level and it can be detected by DCFA-DA fluorescence probe staining. In this study, we found that UVB irradiation increases the intracellular ROS production in HDFa cells. Linalool treatment prevents UVB-mediated ROS production in HDFa cells. Fluorescence microscopic images clearly showed ROS mediated DCF fluorescence in UVB-exposed cells and linalool treatment attenuated this ROS mediated DCF green fluorescence in HDFa cells (Fig 1B).

### Linalool on UVB-induced oxidative damages

**Fig 2:**
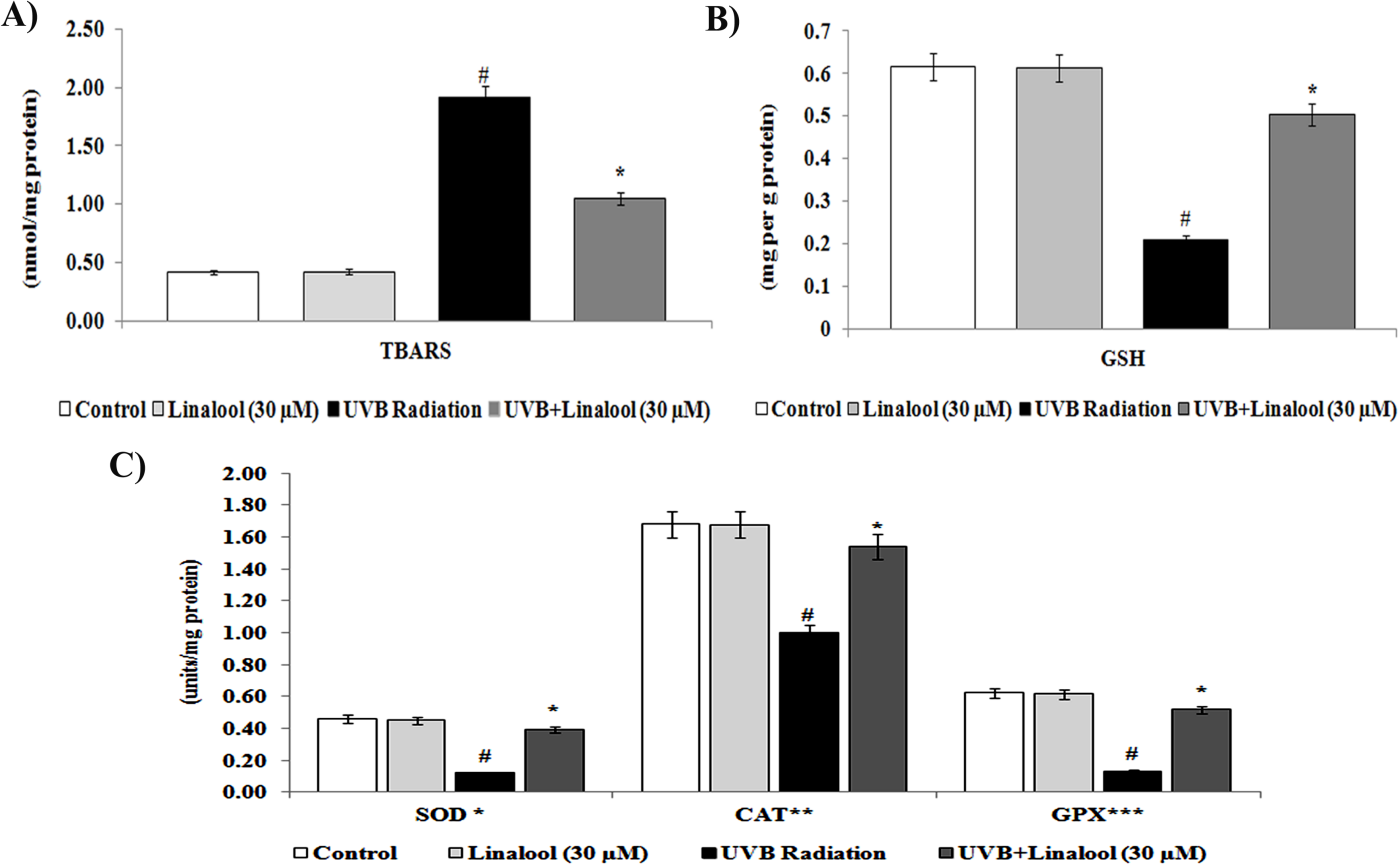
Effect of linalool on UVB-induced lipid peroxidation and antioxidant status in skin cells. (A) Lipid peroxidation status of linalool and/or UVB irradiated HDFa cells. TBARS expressed as nmol/mg protein (B) GSH level of linalool and/or UVB irradiated HDFa cells. GSH level was expressed as mg/g protein (C) Enzymatic antioxidants status of linalool and/or UVB irradiated HDFa cells. *Enzyme concentration required for 50% inhibition of nitroblue tetrazolium reduction in one minute. **μmol of hydrogen peroxide consumed per minute. ***μg of glutathione consumed per minute. Values are given as means ± SD of six experiments in each group. Values not sharing a common marking (# and *) differ significantly at P = 0.05 (DMRT).

Membrane lipids are major targets of UVB-induced oxidative damage. The level of lipid peroxidation in UVB-irradiated and/or linalool treated HDFa cells were determined by analyzing thiobarbituric acid reactive substances (TBARS) (Fig 2A). We observed that an elevated TBARS levels (an indicator of lipid peroxidation) in UVB-exposed dermal cells (Fig 2A). Besides, pretreatment of linalool significantly prevented UVB-induced TBARS formation in UVB-irradiated HDFa.

**Fig 3:**
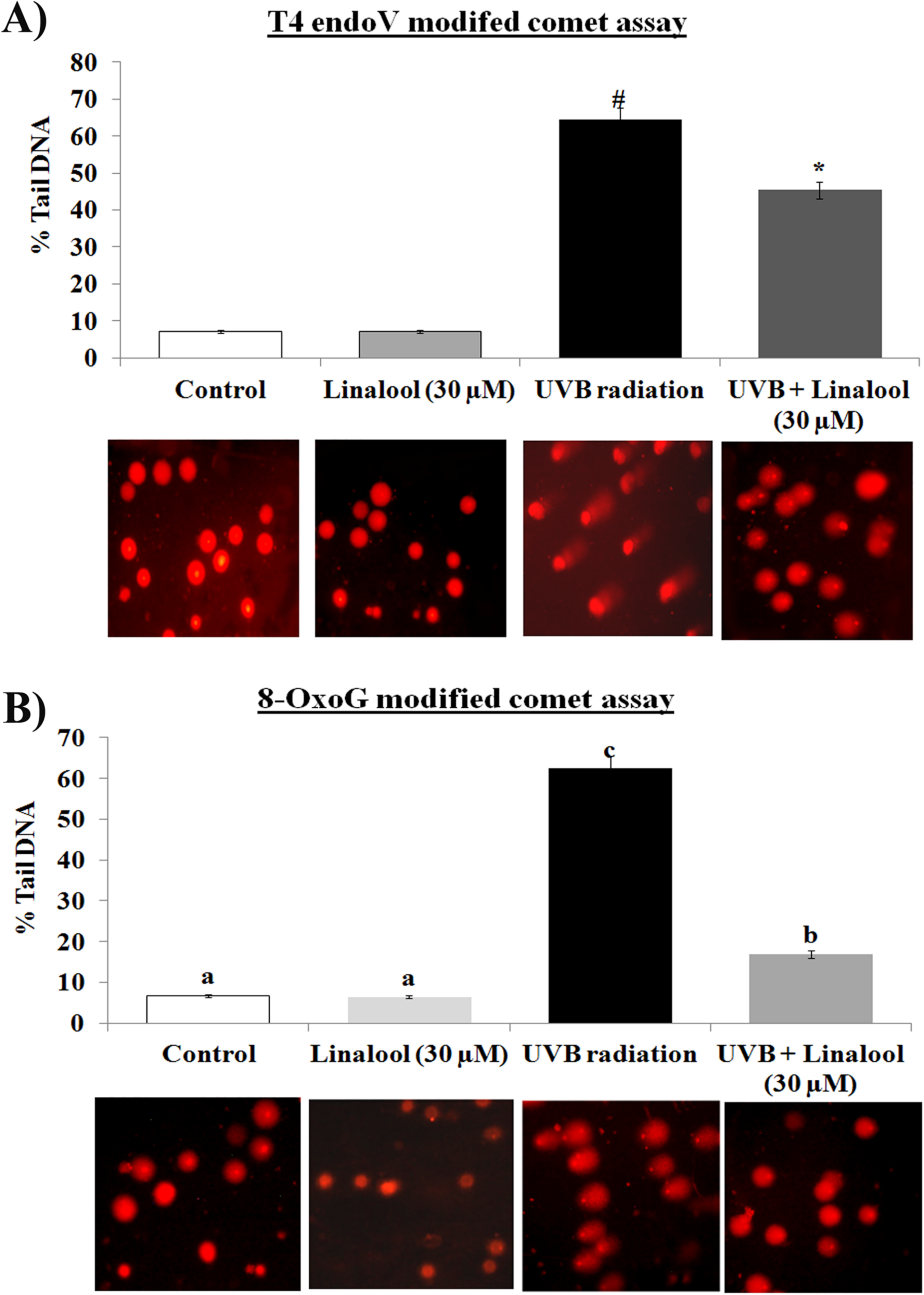
Effect of linalool on UVB induced DNA damage in HDFa cells. (A) Linalool on UVB induced CPDs formation was analyzed by T4 modified comet assay. Fluorescence microscopic images (20X) were recorded using fluorescence microscope (Cell imaging station, life technologies) under red fluorescence lamb. (B) Linalool on UVB induced 8-OxoG was analyzed by 8-OxoG modified comet assay. Fluorescence microscopic images (20X) were recorded using fluoresece microscope (Cell imaging station, life technologies) under red fluorescence lamb. The percentage tail DNA was calculated by CASP software for both CPD and 8-OxoG modified assay. Values are given as means ± SD of six experiments in each group. Values not sharing a common marking (# and *) differ significantly at P = 0.05 (DMRT).

Skin cells have evolved a complex of endogenous antioxidant defence system which may scavenge free radicals and oxidative damage during the UVB-exposure. The enzymatic and non-enzymatic antioxidants in UVB-irradiated and/or linalool treated HDFa cells were determined by biochemical estimations. In this study, reduced glutathione (GSH) level was decreased in UVB irradiated HDFa cells. Whereas, linalool pretreatment prevented UVB induced depletion of GSH levels in HDFa cells (Fig 3B). In addition, we found that a single low dose exposure of UVB irradiation significantly decreased the activities of SOD, CAT and GPx in HDFa. Treatment with linalool prior to UVB irradiation significantly prevented the depletion of SOD, CAT and GPx in HDFa cells (Fig 3C).

### Linalool modulates UVB-mediated DNA damage

UVB radiation induces direct DNA damage resulted in CPDs formation and indirect ROS-mediated DNA damage resulted in 8-OxoG formation. T4endoV modified comet assay analyses for direct CPDs formation and hOGG1 modified comet assay analyses for 8-OxoG formation. In this study, UVB irradiation significantly increases the CPDs formation in HDFa cells when compared to untreated HDFa cells. Treatment with linalool prior to UVB could not able to more significantly prevents the CPDs formation (Fig 3A). Whereas, hOGG1 modified comet assay showed that single UVB irradiation increased 8-OxoG oxidative lesion in HDFa cells (Fig 3B) and which has been prevented by 30 μM of linalool treatment in HDFa cells.

### Linalool on UVB-induced apoptotic responses in HDFa

**Fig 4:**
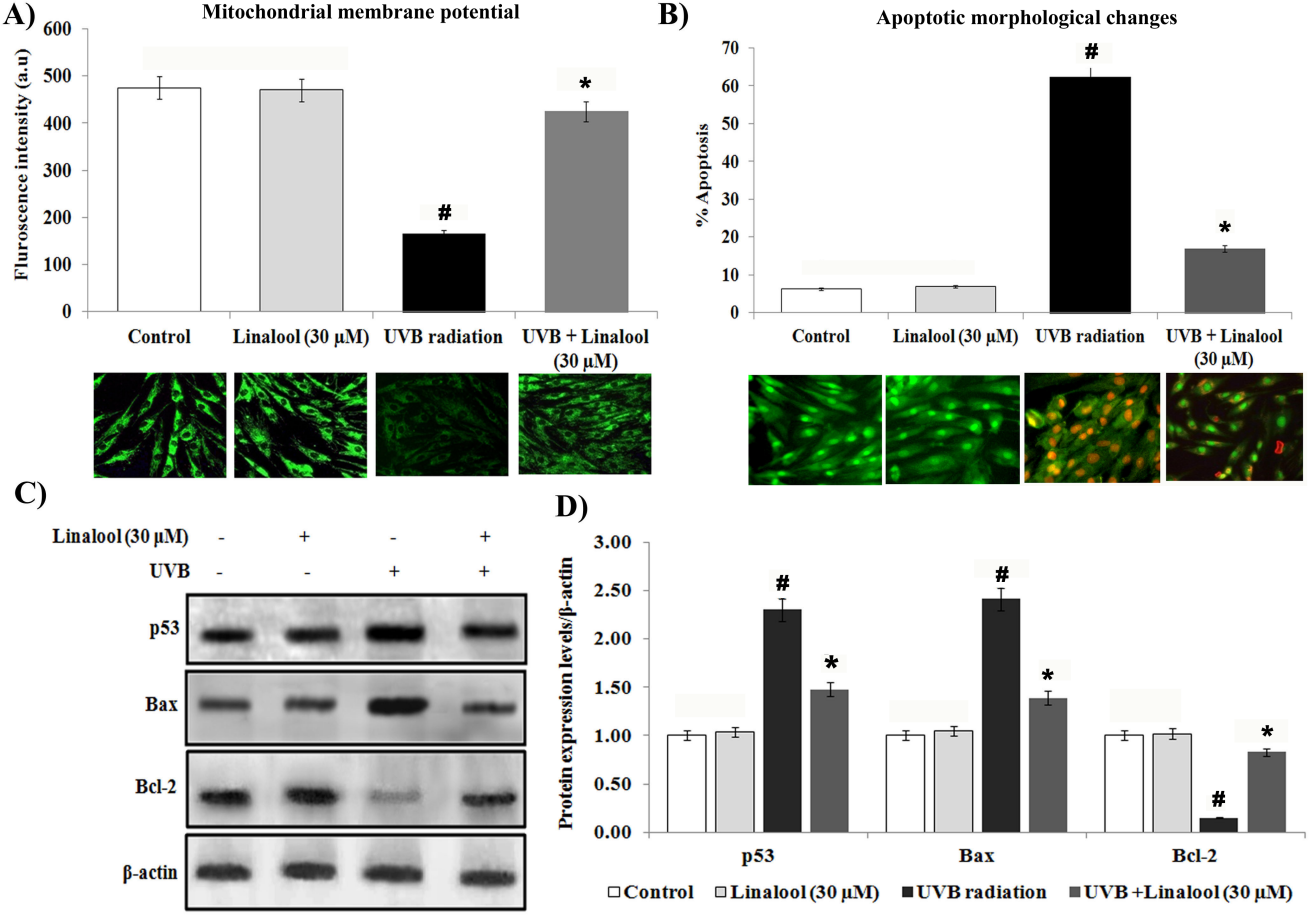
Linalool protects UVB-induced apoptosis in HDFa cells. (A) Linalool prevents UVB induced alteration of mitochondrial membrane potential in HDFa cells. Linalool treated and/or UVB irradiated cells were treated with Rh-123 staining. Fluorescence microscopic images (20X) were recorded using fluoresece microscope (Cell imaging station, life technologies) under green fluorescence lamb. Bar diagram represents fluorescence intensity made with excitation and emission at 485 ± 10 and 530 ± 12.5 nm, respectively using a multimode reader (Teccan, Austria). Data expressed as means ± SD of six experiments in each group. Values not sharing a common marking (# and *) differ significantly at P < 0.05 (Duncan’s multiple-range test). (B) Linalool against UVB induced apoptotic morphological changes were measured by AO/EtBr staining. Linalool treated and/or UVB irradiated cells were treated with AO/EtBr staining, fluorescence microscopic images (20X) were recorded and % apoptotic cells were calculated. (C) Effect of linalool on UVB-induced expression of apoptotic markers in HDFa. Western blot analysis of Bax, p53 and Bcl-2 expression in HDFa. (D) Band intensities were analyzed by Image studio software and normalized to ß-actin level. Values are given as means ± SD of six experiments in each group. Values not sharing a common marking (# and *) differ significantly at P < 0.05 (DMRT).

Alteration of mitochondrial membrane potential has been considered to be an early sign of apoptosis. The alteration in the mitochondrial membrane potential has been traced by Rhodamine 123 staining. In this study, control and linalool alone treated HDFa cells showed significant fluorescence intensity due to accumulation of rhodamine-123 in polarized mitochondria (Fig 4A). We observed that UVB-irradiation altered mitochondrial polarization. Whereas, linalool treatment prevented UVB-induced loss of mitochondrial polarization in skin cells.

Apoptotic morphological changes were analyzed by ethidium bromide (EtBr) and acridine orange (AO) staining to differentiate cells that are apoptotic and/or viable. There was an increased apoptotic feature i.e nuclear condensation, membrane blebbing and higher incidence of EtBr stained cells in UVB irradiated HDFa (Fig 4B). Linalool pretreatment prevented UVB-induced apoptotic features in HDFa cells. Control and linalool alone treated cells showed intact nuclear, and green fluorescence stained cells.

Further, proapoptotic and antiapoptotic markers expression were analyzed by western blotting. Our result shows that single low-dose UVB-exposure caused significant overexpression of proapoptotic proteins such as Bax and p53 and downregulation of antiapoptotic protein, Bcl-2 in skin cells (Fig 4C and 4D). Conversely, linalool pretreatment significantly prevented UVB-mediated overexpression of proapoptotic markers, Bax and p53 and downregulation of antiapoptotic protein, Bcl-2 expression HDFa cells.

### Linalool inhibits UVB-induced MAPKs and NF-κB expression in HDFa

**Fig 5:**
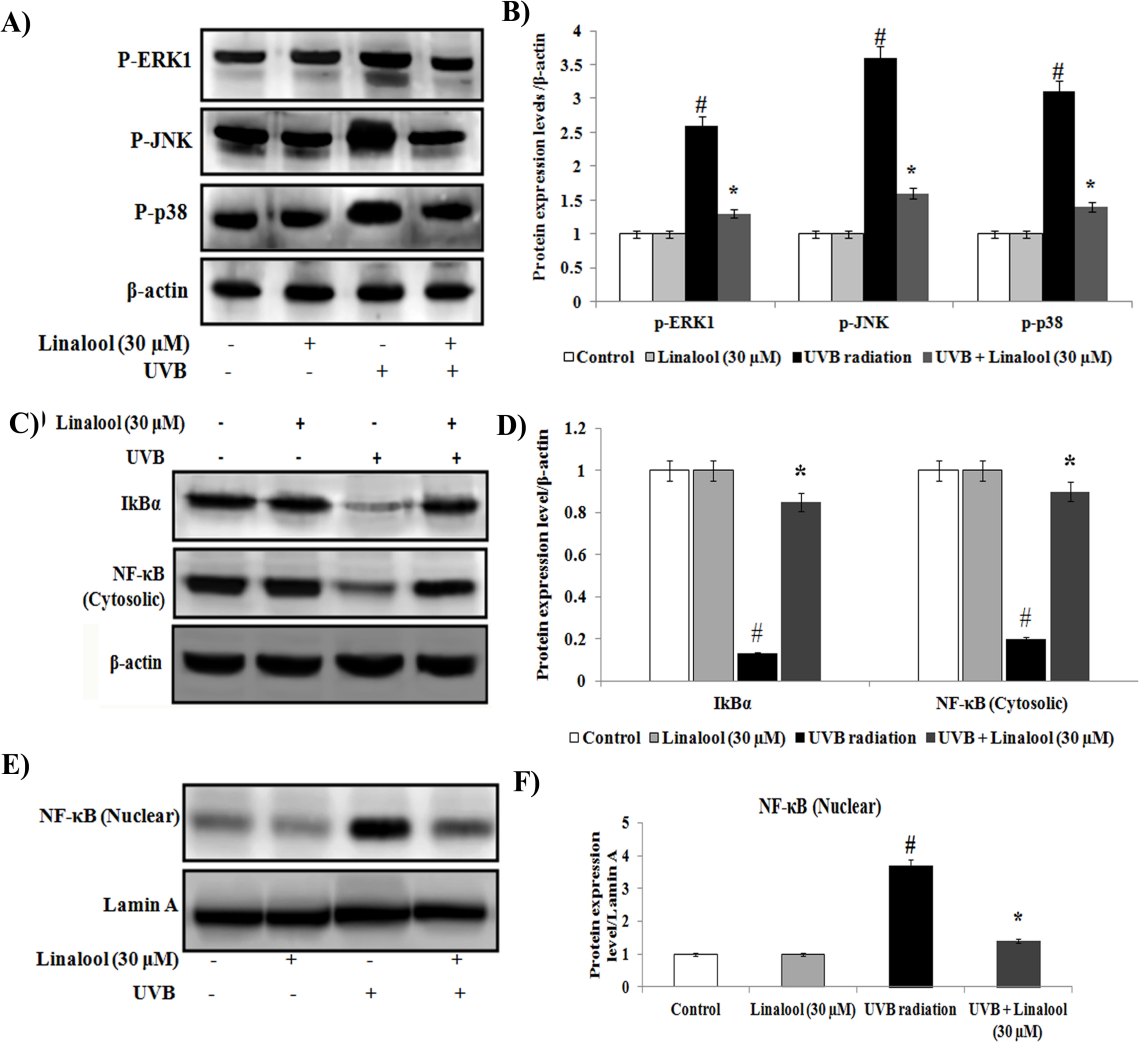
Effect of linalool on UVB-induced MAPKs and NF-κB signaling in HDFa. **(A)** Western blot analysis of p-ERK1, p-JNK and p-p38 expression in HDFa. **(B)** The quantification of protein was performed by densitometric analysis using Image-studio software (LI COR, USA). The densitometry data represent means ± SD from three immunoblots and are shown as relative density of protein bands normalized to ß-actin level. (**C)** Western blot analysis of cytosolic NF-κB and IκBa expression in HDFa. **(D)** Band intensities were analyzed by Image studio software and normalized to ß-actin level. **(E)** Western blot analysis of NF-κB nuclear expression in HDFa. **(F)** Band intensities were analyzed by Image studio software and normalized to Lamin A level. The densitometry data represent means ± SD from three immunoblots and are shown as relative density of protein bands normalized to Lamin A level. Values not sharing a common marking (# and *) differ significantly at P < 0.05 (DMRT).

The MAPK signaling cascades are targets for UVB and are important in the regulation of the multitude of UV-induced oxidative responses. In this study, there was an general trend of overexpression of phosphorylated ERK1, JNK and p38 proteins in UVB-exposed skin cells (Fig 5A and 5B). Whereas, linalool treatment significantly prevented UVB-induced phosphorylation of ERK1, JNK and p38 in HDFa cells.

Further, we observed that translocation of NF-κB from cytosol to nucleus and degradation of cytosolic IκBα in UVB-irradiated cells (Fig 5C–5F). Conversely, pretreatment of linalool prevents NF-κB translocation to the nucleus and maintains sufficient levels of cytosolic IκBα in irradiated skin cells.

### Linalool inhibits UVB-induced inflammatory markers in HDFa

**Fig 6:**
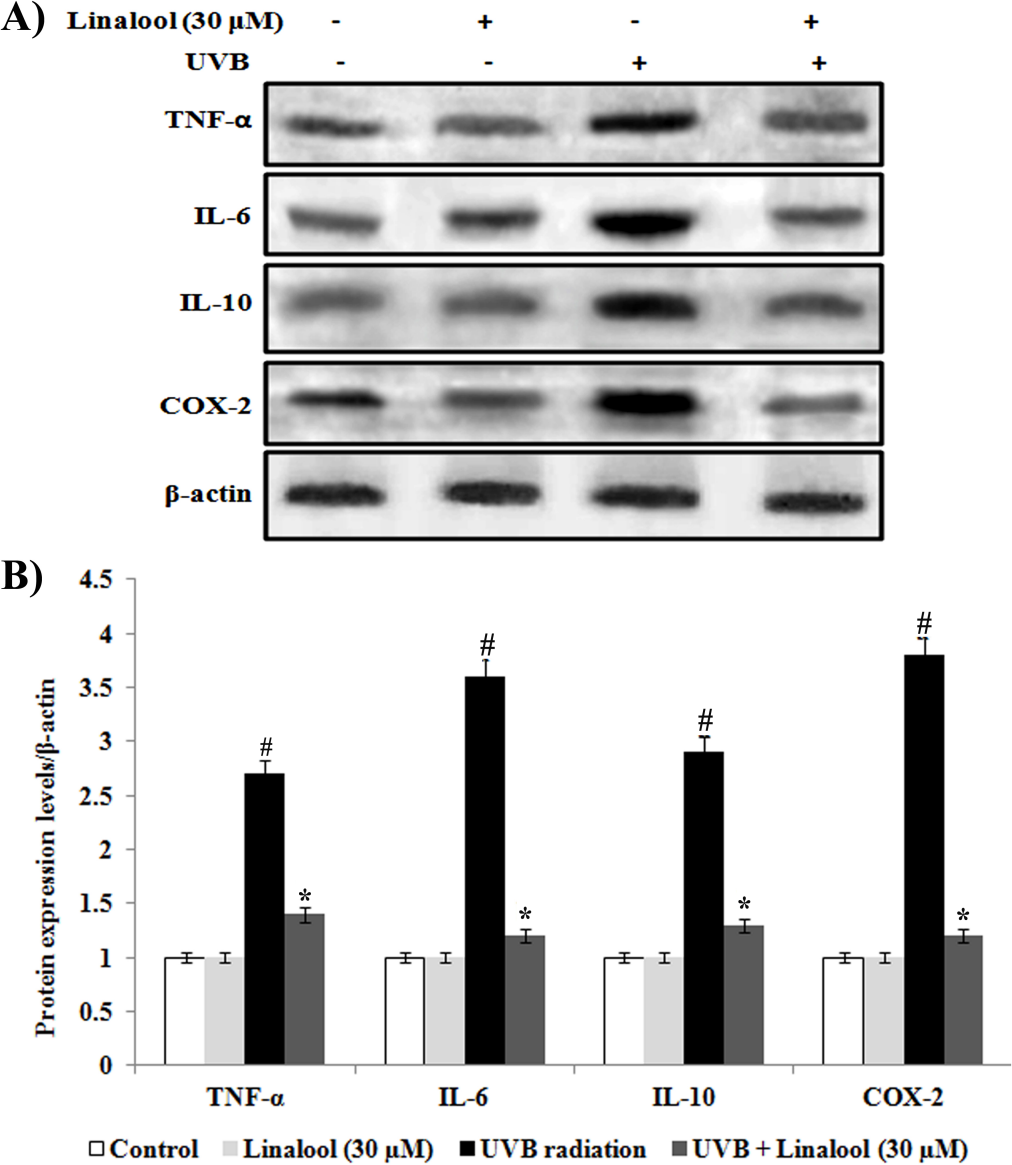
Effect of linalool on UVB-induced inflammatory markers expression in HDFa. (A) Western blot analysis of TNF-α, IL-6, IL-10 and COX-2 expression in HDFa. (B) The quantification of protein was performed by densitometric analysis using Image-studio software (LI COR, USA). The densitometry data represent means ± SD from three immunoblots and are shown as relative density of protein bands normalized to ß-actin level. Values not sharing a common marking (# and *) differ significantly at P < 0.05 (DMRT).

**Fig 7:**
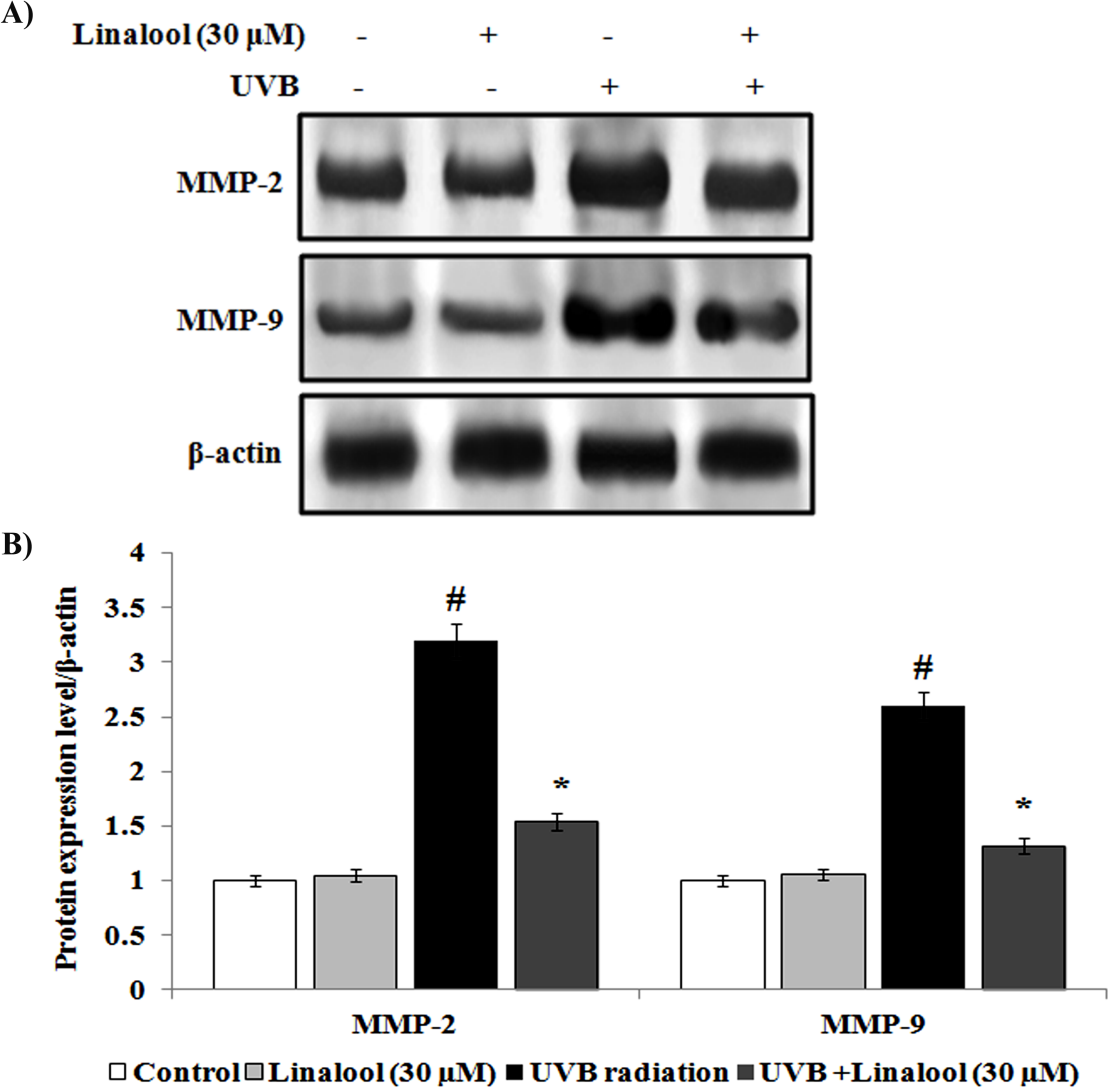
(A) Effect of linalool on UVB-induced expression of matrix metalloproteinases in HDFa. (B) The densitometry data represent means ± SD from three immunoblots and are shown as relative density of protein bands normalized to ß-actin level. Values not sharing a common marking (# and *) differ significantly at P < 0.05 (DMRT).

Overexpression of cytokines and inflammatory proteins are involved in the skin inflammation during the exposure of UVB. We studied the effect of linalool against UVB-induced inflammatory markers expression using western blotting analysis. Single UVB-exposure caused overexpression of TNF-α, IL-6, IL-10 and COX-2 in HDFa cells (Fig 6A and 6B). In contrast, linalool pretreatment significantly inhibits UVB-mediated overexpression of TNF-α, IL-6, IL-10, and COX-2 in skin cells. Matrix metalloproteinases (MMPs) are zinc dependent endopeptidases which are involved in the degradation of extracellular matrix and collegen degradation. In this study, MMP-2 and MMP-9 was significantly overexpressed in UVB-irradiated skin cells when compared to non-exposed skin cells (Fig 7A and 7B). Whereas, linalool treatment prior to UVB-exposure prevented MMP-2 and MMP-9 overexpression in skin cells.

**Fig 8:**
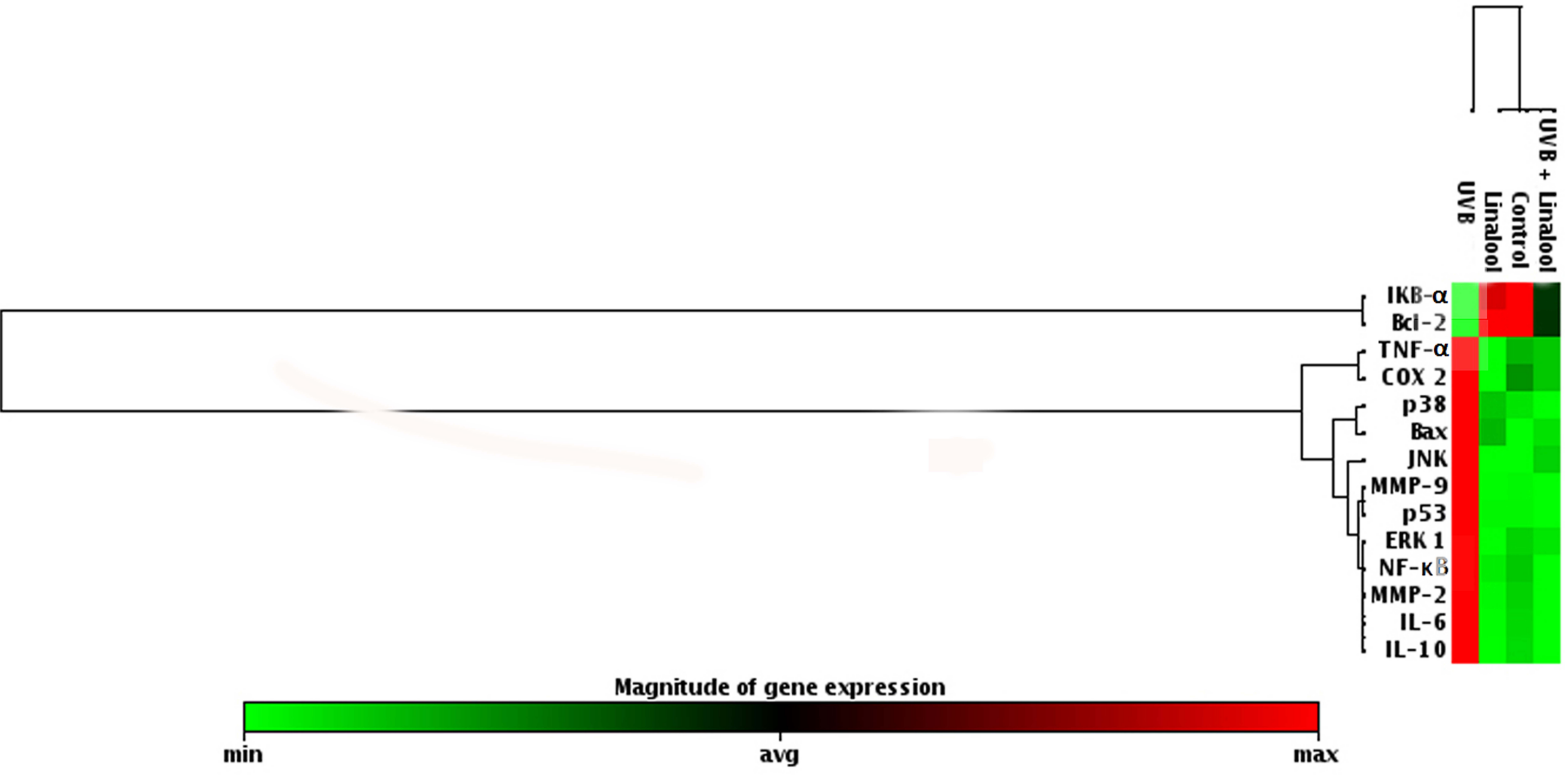
Effect of linalool on UVB-mediated mRNA expression level of oxidative stress marker in HDFa. Cells were treated with linalool following UVB radiation total cellular RNA was isolated and reverse transcribed. The mRNA levels of genes involved in oxidative stress were detected using custom PCR array by following the manufacturer’s instructions. The clustergram results were analyzed by SA Biosciences’ online tool.

Fig 8. show the effect of linalool on the mRNA expression pattern of genes involved in UVB-induced MAPKs, NF-κB, inflammation, apoptosis and photoaging gene expressions in HDFa cells. The results showed there was an increased fold expression of ERK1, JNK, p38, NF-κB, TNF-α, IL-6, IL-10, COX-2, Bax, p53, MMP-2 and MMP-9 and decreased expression of IκBα and Bcl-2 in UVB-irradiated skin cells. Conversely, treatment with linalool significantly decreased fold expressions of ERK1, JNK, p38, NF-κB, TNF-α, IL-6, IL-10, COX-2, Bax, p53, MMP-2 and MMP-9 and an increased mRNA expression of IκBα and Bcl-2 in UVB-irradiated skin fibroblasts.

## Discussion

Exposure of UVB radiation induces intracellular oxidative stress and elicits several cellular signaling. In this study, we evaluated the role of linalool against oxidative stress activated protein kinases in single UVB-exposed human skin cells. Several studies have been reported that various antioxidant agents could protect UVB-induced cell toxicity or cell damage [24]. Linalool is one of the most abundant naturally existing monoterpene in many plant aromatic species and it is a potent antioxidant in nature. In this study, we tested (20–50 μM) concentration of linalool against UVB induced cytotoxicity in HDFa cells. We found that 30 μM of linalool pretreatment significantly prevented UVB-induced cytotoxicity in HDFa cells (Fig 1A). We observed that lower concentration of linalool (20 μM) was insufficient to protect UVB toxicity and the maximum concentrations of linalool (40–50 μM) exhibits adverse results due to cytotoxic nature of linalool at physiologically higher concentrations and hence we chosen 30 μM linalool as optimum protective dose against UVB experiments.

UVB-radiation elicits the induction of ROS levels in the skin cells. UVB-rays interact with cellular photosensitizers like cytochromes, porphyrins etc., resulting in the production of ROS [25]. Elevated ROS levels can induce severe oxidative damages which can induce inflammation and apoptosis [26]. In this study, linalool pretreatment prevented UVB-mediated ROS production in skin cells. Linalool has been reported as an ideal reducing agent because of several of its properties, including (a) the ability to scavenge ROS, (b) the ability to regenerate other antioxidants from their radical or inactive forms [27]. Furthermore, we observed increased TBARS levels in UVB-irradiated cells with concomitant decrease of SOD, catalase and GPX activities. Lipid peroxidation and loss of antioxidants generated by UVB-radiation has been known to be potentially deleterious for cellular function, having cytotoxic effects, cell membrane damage and apoptosis [28]. Monoterpenes are powerful antioxidants and alleviated free radical mediated alterations in several in vitro and in vivo models [29]. Our results demonstrate that linalool prevents UVB-induced lipid peroxidation and loss of antioxidants depletion. Previously, linalool has been proved as a potent inhibitor of lipid peroxidation on DMH induced by colon carcinogenesis and prevents oxidative damage [27].

The UVB radiation induces direct DNA damage through cyclobutane pyrimidine dimers (CPDs) and indirectly induces DNA damage by formation of ROS mediated 8-OxoG which is responsible for majority of mutations [30]. We found that linalool inhibits 20 mJ/cm^2^ of UVB induced 8-OxoG, whereas, linalool was found to be ineffective to inhibits UVB-induced CPD formation in HDFa cells. Antioxidant potential of linalool might contribute for the prevention of UVB induced 8-OxoG formation. Therefore, we subsequently studied the effect of linalool against UVB-radiation induced oxidative signaling and inflammatory responses in HDFa cells.

UVB-induced oxidative DNA damage triggers associated with the expression of several signaling elements [31]. UVB-induced phosphorylation and subsequent activation MAPKs has been shown to induce several transcriptional factors which lead to regulation of inflammation, cell proliferation, aging and carcinogenic events [32]. In this study, phosporylated ERK, p38 and JNK have been observed as key factor for inducing NF-κB expression in irradiated cells. Linalool inhibits UVB-induced phosphorylation of ERK, JNK and p38 in HDFa cells. Similarly, glycyrrhizic acid inhibits ROS mediated photodamage through ER stress and MAPK in UVB-irradiated human skin cells [33]. UVB-induced oxidative DNA damage triggers the activation of NF-κB through MAPK pathway [33]. Once activated by UVB-radiation, the NF-κB unit p65 dissociates from its inhibitory protein IκBa and translocates from the cytoplasm to the nucleus where they trigger the transcription of genes involved in inflammation (TNF-α, IL-6 and IL-10) and photoaging (MMP-2 and MMP-9) [34]. These signaling molecules further negatively alter the function of skin cells, and undesirably impact the metabolism of skin, connective tissue by inducing inflammatory responses and photoaging [35]. Studies also demonstrated that increased production of the immunosuppressive cytokines, IL-10, has been reported in the cutaneous tissue after exposure to UVB [36]. In this study, NF-κB activation was blocked by linalool in HDFa probably through scavenging ROS generation, oxidative DNA damage which subsequently reflected as downregulation of TNF-α, IL-6 and IL-10, MMP-2 and MMP-9.

Further, UVB induced NF-κB also positively triggers p53 activation in the cellular milieu. Acute UVB-radiation induced apoptotic cell death. A major downstream regulation of the apoptotic death signal resides with the Bcl-2/Bax gene family [37]. In this study, single UVB-exposure induces p53 expression and subsequent overexpression of Bax and downregulation of Bcl-2 in skin cells. We found that linalool treatment prevents UVB-induced apoptotic signaling probably through preventing DNA damage and subsequent apoptotic activation. Similarly, melatonin and its metabolites ameliorate UVB-induced damage in human epidermal keratinocytes [38]. Melatonin and its metabolites scavenge both ROS/RNS through a free radical scavenging cascade and exhibit antiproliferative and prodifferentiation properties in human epidermal keratinocytes [39–41].

## Conclusion

**Fig 9:**
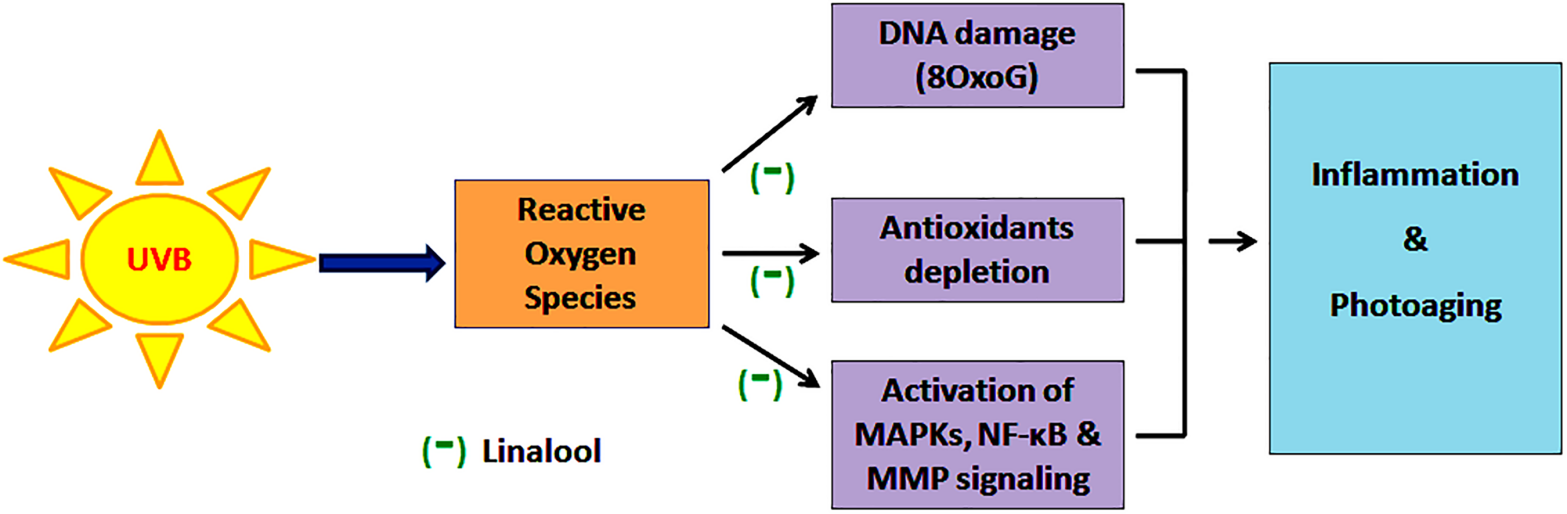
Linalool prevents (-) UVB-induced ROS mediated DNA damage, antioxidant depletion and subsequently MAPKs and NF-κB signalling and thereby prevents oxidative stress, inflammation and photoaging in HDFa cells.

In this study, linalool exhibits significant antioxidant potential in the *in vitro* free radical scavenging system which has been reflected in its preventive effect against low-dose UVB-induced ROS generation and subsequent antioxidant enzyme depletion. Further, linalool prevents UVB-mediated oxidative DNA damage and ineffective against UVB-induced CPDs formation. This indicates its ability to prevent UVB-induced ROS formation and to maintain redox homeostasis in the cell. As linalool prevents UVB induced ROS mediated damages it contributes for the prevention of UVB-induced activation of MAPKs mediated inflammatory reactions (Fig 9). This has been noticed in UVB-induced overexpression of MAPK and NF-κB signaling. Further, it prevents UVB-mediated translocation of NF-κB from the cytosol to the nucleus. Moreover, linalool protects skin cells from UVB-induced photoaging responses through modulating expression of inflammatory cytokines and MMPs. Thus, linalool prevents oxidative stress activated protein kinases in single low-dose UVB-exposed human skin cells. Blockade of the oxidative stress signaling elements may offer an effective approach to prevent skin damage resulting from acute solar radiation.

## Materials and methods

### Chemicals

Fetal bovine serum (FBS), fibroblast growth factor, heparin and trypsin-EDTA were procured from Invitrogen Bioservices, India. Linalool, monoclonal antibodies such as p-ERK1, p-JNK, p-p38, IκBα, NF-κB, TNF-α, IL-6, IL-10, COX-2, Bax, Bcl-2, p53, MMP-2, MMP-9,β-actin, lamin-A and goat anti-mouse IgG-HRP polyclonal antibody, 3-(4, 5-dimethyl-2-thiaozolyl)-2,5-diphenyl-2H tetrazolium bromide (MTT), 2,7-diacetyl dichlorofluorescein (DCFH-DA) were purchased from Sigma chemical Co., St. Louis, MO, USA.

### Human dermal fibroblasts cell culture

The human dermal fibroblasts (HDFa) cells were procured from Invitrogen Bioservices, India. The HDFa cells were cultured at 37°C in 5% CO_2_ in DMEM medium supplemented with LSSG kit (fetal bovine serum, 1 μg/ml, basic fibroblast growth factor 3 ng/ml, heparin 10 ng/ml and antibiotics). The HDFa cells were maintained to grow for 7 days to reach the maximum confluence and cells in between 2–6 passages were used for all the experiments. After reaching 80–90% confluence cells were sub-cultured and used for further experiments.

### Irradiation procedure

The HDFa cells were treated with desired concentrations of linalool (30 μM) and incubated in serum-free medium for 1 h at 37°C in a 5% CO_2_ environment. After washing with PBS, the cells were exposed to UVB-radiation using Philips TL40W/12 RS lamp (Heber Scientific), emitting 312 nm and an intensity of 2.2 m W/cm^2^ for 9 min under a thin layer of PBS. The exposure of UVB was controlled by the dosimeters regulating system. In this system, dose units could be performed in mJ/cm^2^ for UVB nm; variations in energy output are automatically compensated to deliver the desired dose. The filters present in the instrument enables us to irradiate the cells to 312 nm of UVB radiation. To completely block UVC radiation from the lamp, the cell culture plates were covered with 295 nm cut-off cellulose foil filter during the irradiation. After irradiation, cells were again washed using PBS and incubated for 6 h, then harvested for the analysis of biochemical and molecular changes. The sham-exposed cells were treated equivalently, except that the cells were covered with aluminum foil to block radiation.

### MTT based cytotoxicity assay

Linalool against UVB induced cell toxicity was analyzed by MTT assay. Cultured fibroblasts were seeded (1×10^6^ cells/ml) into 96 well plates for 24 h. Then, the HDFa cells were treated with desired concentrations of linalool (20–50 μM) and incubated in serum-free medium for 1 h at 37°C in a 5% CO_2_ environment. After washing with PBS, the cells were exposed to UVB-radiation in a thin layer of PBS and cultured in the presence of 5% CO_2_ at 37°C for 24 h. The MTT (0.5 mg/ml) was added and then again incubated for another 4 h, centrifuged and 200 μl dimethyl sulfoxide (DMSO) was added into each tubes. Absorbance (OD) was measured in a microplate reader at 560 nm.

### Free radical scavenging assays

Superoxide anion scavenging ability of linalool was analyzed by the method of Nishimiki et al., 1973[42] with modifications. Oxidation of NADH by phenazine methosulphate (PMS) to PMS_red_ and conversion of oxidized nitroblue tetrazolium (NBT_oxi_) to the reduced NBT_red_, which form a violet colour complex, was observed. Ascorbic acid was used as standard for the comparison of superoxide anion scavenging property.

The hydroxyl radical scavenging ability of linalool was analyzed as per Halliwell et al., 1987 [43]. Hydroxyl radicals are produced by the reduction of H_2_O_2_ from transition metal ions with ascorbic acid. DMSO was used as standard for the comparison hydroxyl radical scavenging activity.

### Intracellular ROS measurement

Intracellular ROS generation was measured by non-fluorescent probe, 2,7-diacetyl dichlorofluorescein (DCFH-DH) that can penetrate into the intracellular matrix of cells, where it is oxidized by ROS to fluorescent dichlorofluorescein (DCF) [44]. Briefly, an aliquot of isolated cells (8 × 10^6^ cells/ml) were made up to a final volume of 2 ml in normal phosphate buffered saline (pH 7.4). 1 ml aliquot of cells was taken to which 1 μl of DCFH-DA (1 mg/mL) was added and incubated at 37°C for 30 min under dark condition. Fluorescent measurements were made with excitation and emission at 485 ± 10 and 530 ± 12.5 nm, respectively using a multimode reader (Teccan, Austria). Images were taken on an epifluorescent microscope (Nikon, Eclipse TS100, Japan) with a digital camera (Nikon 4500 Coolpix, Japan).

### Lipid peroxidation measurement

Lipid peroxidation status was analyzed by thiobarbituric acid reactive substance (TBARS) as per the method of Niehaus and Samuelsson 1968 [45]. Linalool and/or UVB treated HDFa cell lysates at the volume of 0.2 ml was added with 0.2 ml of 8.1% SDS and 3 ml of TBA reagent (equal volumes of 0.8% TBA and 20% acetic acid, pH 3.5). Total volume was made up to 4 ml with distilled water and kept at 95°C for 1 h in a water bath. The chromogen was extracted with *n* -butanol and pyridine (15:1 v/v). The absorbance was measured at 530 nm by using multimode reader. TBARS were expressed in mM/mg proteins.

### Antioxidants status analysis

The activities of enzymatic and non-enzymatic antioxidants were assessed by colorimetry. Superoxide dismutase (SOD) activity was measured as per the method of Kakkar et al., 1984 [46], based on inhibition of formation NADH-PMS-NBT complex. Catalase (CAT) activity was analyzed by measuring the H_2_O_2_ after incubating with dichromate-acetic acid solution as per the method of Sinha, 1972 [47]. The activity of glutathione peroxidase (GPx) was assayed by the method of Rotruck et al., 1972 [48]. The GSH level was measured by the method of Ellman, 1959 [49].

### Measurement of 8-deoxy guanosines (8-OxoG) and cyclobutane pyrimidine dimers (CPD)

Modified alkaline single cell gel electrophoresis (comet assay) was used to detect 8-OxoG and CPD [50–53]. A layer of 1% NMPA was prepared on microscope slides. After UVB-irradiation, HDFa cells (50 μl) were mixed with 200 μl of 0.5% LMPA. The suspension was pipetted onto the precoated slides. Slides were kept in cold lysis solution at pH 10 (2.5 M NaCl, 100 mM Na2EDTA, 10 mM Tris pH 10, 1% Triton X-100, 10% DMSO) and kept at 4°C for 60 min. Immediately after lysis, the slides were incubated for 30 min with 40 unit of T4endoV for CPD determination and 0.10 unit per gel of hOGG1 for 8-OxoG determination. To allow DNA unwinding, the slides were placed in alkaline electrophoresis buffer at pH 13 and left for 25 min. Subsequently, the slides were transferred to an electrophoresis tank with fresh alkaline electrophoresis buffer and the electrophoresis was performed at field strength of 1.33 V/cm for 25 min at 4°C. Slides were neutralized in 0.4 M Tris (pH 7.5) for 5 min and stained with 20 μg/ml ethidium bromide. Comet observations were made using a 40× objective using an epifluorescent microscope. Comet attributes were analyzed using CASP software. Each slide was divided into five regions; in each region at least 10 cells were randomly selected and analyzed (approximately 50 cells/slide). Six experiments were made for each groups.

### Changes in mitochondrial transmembrane potential and apoptotic morphological changes

The changes in *Δ*ψm in different treatment groups were observed microscopically and determined spectrofluorometrically using Rhodamine-123 staining. Treated cells (UVB-irradiated and/or linalool) stained with 1 μl of rhodamine-123 (5 mmol) was added and kept in the incubator for 30 min as previously described [54]. The cells were then washed with PBS and observed with a fluorescence microscope using blue filter (450–490 nm).

Acridine orange and ethidium bromide method was used to differentiate condensed apoptotic nuclei from normal cells [55]. Treated cells were stained with 1:1 ratio of dyes and observed under a fluorescence microscope with a magnification of 20× the number of cells showing apoptotic morphology was counted and compared to total number of cells present in the field.

### Preparation of total cell lysate, cytosolic and nuclear extraction

After treatment of linalool and/or UVB irradiation, HDFa cells were harvested and washed with PBS. Whole cell lysates were prepared in a RIPA lysis buffer (120 mM NaCl, 40 mM Tris pH 8.0, 0.1% NP40) containing 1% β-mercaptoethanol, 0.1 M Na_3_VO_4_, 0.5 M NaF and protease inhibitor cocktail and then centrifuged (13000 g for 15 mins). Supernatants were collected and it was used as total cell lysates.

In addition, cytosolic and nuclear extracts were prepared by using appropriate buffer. Briefly, the cells were pelleted, resuspended in 100 μL of cytosolic buffer (10 mM HEPES, pH 7.9, 1.5 mM MgCl_2_, 10 mM KCl, 0.1 mM EDTA, 0.1 mM EGTA, 1 mM DTT, 0.5 mM PMSF, 10 μg/mL leupeptin, 10 μg/mL aprotinin, 1 mM Na_3_VO_4_, 1 mmol·L−1 NaF) and kept on incubation for 15 min at ice bucket. Then, the cells were lysed with 6.25 μL 10% Nonidet P-40. After centrifugation (13,000 g for 5 min), the supernatant was collected as cytosolic extract and the pellet was resuspended in 12.5 μL nuclear buffer (20 mM HEPES, pH 7.9, 400 mM NaCl, 1 mM EDTA, 1mM EGTA, 1mM DTT, 1mM PMSF,5 μg/mL leupeptin, 5 μg/mL aprotinin) on ice for 30 min. After centrifugation (13,000 g for 15 min) at 4°C, the supernatant was collected as nuclear extract. Further, the total cell lysates, cytosolic and nuclear extracts were concentrations were measured by the Nanodrop (Thermo Scientific). Aliquots of the lysates (50 μg) were boiled for 5minutes and then subjected to electrophoresis on a 10% sodium dodecyl sulfate polyacrylamide gel.

### Western blot analysis

Protein lysates (cytosolic and nuclear fraction) were separated by SDS polyacrylamide gel electrophoresis (PAGE) and transferred to PVDF membranes (Bio-Rad) for the analysis of MAPKs, NF-κB, TNF-α, IL-6, IL-10, COX-2, Bcl-2, Bax, p53, MMP-2, and MMP-9. Membranes were washed with Tris-buffered saline (TBS) containing 0.05% Tween 20 (TBST) and were blocked using TBS containing 5% BSA. The membranes were then washed with TBST and then kept overnight at 4°C with appropriate primary antibodies. Membranes were washed and treated with HRP-conjugated secondary antibodies for 1 h at room temperature. The PVDF membranes were then washed thrice with 10 min interval; bands were developed and detected using a chemiluminescence substrate. The images were acquired by Image Studio software (LI-COR).

### RNA isolation and PCR array

Total RNA was isolated from UVB and linalool-treated HDFa cells using the RNeasy mini kit (Qiagen, India) according to the manufacturer’s instruction and stored at -80°C until used for gene expression analysis by qRT-PCR arrays. The purity of isolated RNA was measured by the Nanodrop (Thermo Scientific). Total RNA with an A260/A280 ratio >1.9 and no detectable contamination of DNA (PCR) were employed for the gene-expression analysis.

cDNA was synthesized using 5 μg total RNA by First Strand cDNA Synthesis Kit (Qiagen reverse transcriptase kit). The cyclic conditions were followed for cDNA synthesis, 25°C for 10 min, 42°C for 50 min and 75°C for 15 min. The relative expression of ERK1, JNK, p38, IκBα, NF-κB, TNF-α, IL-6, IL-10, COX-2, Bax, Bcl-2, p53, MMP-2 and MMP-9 were observed by PCR array using RT^2^ real-time SYBR Green/Rox PCR master mix (Qiagen) on eppondrof real plex. 500 ng of cDNA was amplified in 20 μl total volume containing Taq polymerase enzyme and oligonucleotides were added on the primer coated PCR array 96 well plates. Cycling conditions were as followed for DNA amplification, 2 min initial denaturation at 95°C followed by 35 cycles with 15 s. denaturation at 94°C, 30 s primer annealing at 58°C and 30 s of extension at 72°C. For quantitation, differences between treatments were analyzed by comparing mRNA levels to the control after normalization to GAPDH mRNA levels. The fold changes were plotted as clustergram using SA Biosciences analysis online tool.

### Statistical analysis

All the values were expressed as means of six (n = 6) for antioxidant determinations. The data were statistically analyzed by one-way analysis of variance (ANOVA) and the group means were analyzed by Duncan’s multiple range test (DMRT). The results were considered significant if the P value is the ≤ 0.5 levels.

## Supporting information

S1 Data Data analysis for Fig 1. (XLSX)

S2 Data Data analysis for Figs 2 and 3. (XLSX)

S3 Data Data analysis fir Figs 4 and 5. (XLSX)

S4 Data Data analysis for Figs 6 and 7. (XLSX)
